# Characterization of Simple Sequence Repeats (SSRs) in Ciliated Protists Inferred by Comparative Genomics

**DOI:** 10.3390/microorganisms8050662

**Published:** 2020-05-01

**Authors:** Yuan Li, Xiao Chen, Kun Wu, Jiao Pan, Hongan Long, Ying Yan

**Affiliations:** Institute of Evolution & Marine Biodiversity, KLMME, Ocean University of China, Qingdao 266003, China; ly2722@stu.ouc.edu.cn (Y.L.); seanchen607@gmail.com (X.C.); wukun@stu.ouc.edu.cn (K.W.); panjiao@stu.ouc.edu.cn (J.P.)

**Keywords:** evolution, genome instability, genome repetivity, protists, simple sequence repeats

## Abstract

Simple sequence repeats (SSRs) are prevalent in the genomes of all organisms. They are widely used as genetic markers, and are insertion/deletion mutation hotspots, which directly influence genome evolution. However, little is known about such important genomic components in ciliated protists, a large group of unicellular eukaryotes with extremely long evolutionary history and genome diversity. With recent publications of multiple ciliate genomes, we start to get a chance to explore perfect SSRs with motif size 1–100 bp and at least three motif repeats in nine species of two ciliate classes, Oligohymenophorea and Spirotrichea. We found that homopolymers are the most prevalent SSRs in these A/T-rich species, with AAA (lysine, charged amino acid; also seen as an SSR with one-adenine motif repeated three times) being the codons repeated at the highest frequencies in coding SSR regions, consistent with the widespread alveolin proteins rich in lysine repeats as found in *Tetrahymena*. Micronuclear SSRs are universally more abundant than the macronuclear ones of the same motif-size, except for the 8-bp-motif SSRs in extensively fragmented chromosomes. Both the abundance and A/T content of SSRs decrease as motif-size increases, while the abundance is positively correlated with the A/T content of the genome. Also, smaller genomes have lower proportions of coding SSRs out of all SSRs in *Paramecium* species. This genome-wide and cross-species analysis reveals the high diversity of SSRs and reflects the rapid evolution of these simple repetitive elements in ciliate genomes.

## 1. Introduction

Simple sequence repeats (SSRs), also known as tandem repeats, are abundant components present in all known genomes. They are major contributors of genome repetivity and are associated with transposable elements [1,2,3,4]. Homopolymer runs and microsatellites are two well-known representatives of SSRs. These repeats are usually insertion/deletion (indel) mutation hotspots that cause replication slippage of DNA polymerases. They could lead to high genome instability thus causing certain diseases, for example Lynch syndrome, a hereditary non-polyposis colorectal cancer in humans [5,6,7,8]. The high indel mutation rate of SSRs increases genetic variation between individuals in a population, making SSRs suitable tools for developing genetic markers and for studies of population genetics in a variety of organisms; tandem repeats of amino acids may also facilitate rapid generation of morphological variation [9,10,11,12,13,14].

Ciliates are microbial eukaryotes with high species and genomic diversity, and are characterized by nuclear dimorphism [15,16,17,18,19,20,21,22,23]. The macronucleus is transcriptionally active whereas the micronucleus is only active during sexual reproduction [24]. Genomes of these unicellular organisms are highly A/T-rich and repetitive, causing difficulties in genome-sequencing. Nonetheless, genomes have been deciphered for increasing numbers of species, thus providing the opportunity to study genome evolution using comparative genomics methods [15,25,26,27,28,29,30,31,32].

During development of the new macronucleus, most micronuclear non-coding sequences, including repetitive ones, are eliminated, while some long repeats are still retained in macronuclear genomes [15,33,34]. It remains a question how the genome rearrangement process changes the shape and span of the frequency distribution of macronuclear SSRs, compared with that of the micronucleus.

In this study, we explore the genome-wide variation of SSR characteristics using published high-quality genomes of nine ciliates: *Ichthyophthirius multifiliis*, *Oxytricha trifallax*, *Paramecium biaurelia*, *P. caudatum*, *P. sexaurelia*, *P. tetraurelia*, *Pseudocohnilembus persalinus*, *Stylonychia lemnae*, and *Tetrahymena thermophila* (Table 1). We focus on the patterns of distribution, structure, and codons of SSRs, and the evolutionary mechanisms that determine these patterns.

## 2. Materials and Methods

### 2.1. Genome Sequences and Annotations

Genome and annotation data of the following species were downloaded from the National Center for Biotechnology Information (NCBI) Genome database: *Ichthyophthirius multifiliis* (macronucleus: GCF_000220395.1)*, Oxytricha trifallax* (macronucleus: GCA_000295675.1; micronucleus: GCA_000711775.1)*, Paramecium tetraurelia* (macronucleus: GCA_000715435.1)*, Pseudocohnilembus persalinus* (macronucleus: GCA_001447515.1)*, Stylonychia lemnae* (macronucleus: GCA_000751175.1), and *Tetrahymena thermophila* (macronucleus: GCF_000189635.1; micronucleus: GCA_000261185.1). Those of *Paramecium biaurelia*, *P. caudatum*, and *P. sexaurelia* were downloaded from the ParameciumDB database (https://paramecium.i2bc.paris-saclay.fr/; access on 20 February 2020).

### 2.2. Analysis of Simple Sequence Repeats (SSRs)

Perfect SSRs with motif size 1–100 bp (each motif has ≥3 repeats; no SSR with motif size >100 bp was detected in any genomes involved in this study) were detected with a Perl program originally developed by Dr. Way Sung, University of North Carolina, Charlotte. This program applies a greedy algorithm to find the maximum number of repeats. For motifs nested in one SSR, which are rare, only the smallest motif was counted. Details are described in Sung et al. [38]. Codons in SSRs were iterated from coding sequences of each genome, with both the strand and starting codon position taken into account. All statistical tests were carried out in R 3.4.4 [39]. Plotting was performed using R packages ggplot2 and ggpmisc.

## 3. Results

The detailed genomic features of the nine ciliate species are shown in Table 1. All genomes are A/T-rich (A/T content: 68.30%–84.09%; Table 1) with a wide range of genome sizes and total gene numbers. The species belong to one of two ciliate classes: Oligohymenophorea (*Ichthyophthirius multifiliis*, *Paramecium biaurelia*, *P. caudatum*, *P. sexaurelia*, *P. tetraurelia*, *Pseudocohnilembus persalinus*, *Tetrahymena thermophila*) and Spirotrichea (*Oxytricha trifallax*, *Stylonychia lemnae*). Most macronuclear chromosomes in the two spirotricheans are extremely fragmented and amplified during genome rearrangement.

### 3.1. Size Distribution and A/T Content of SSRs

**Figure 1:**
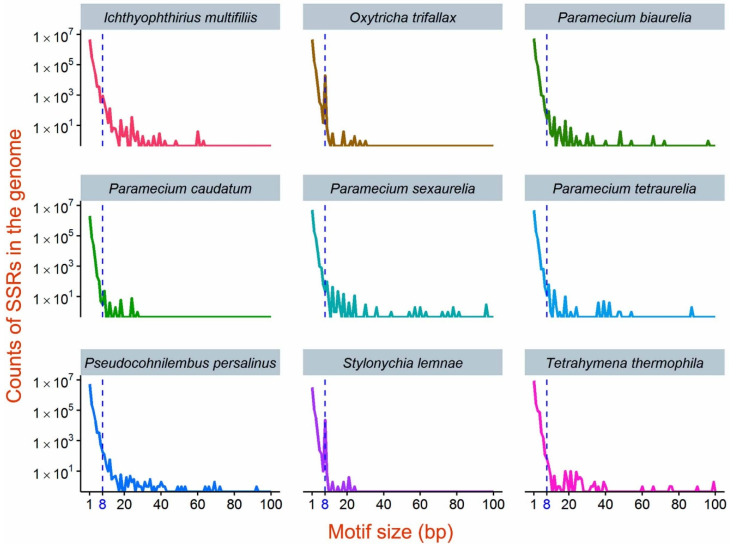
Counts of simple sequence repeats (SSRs) with 1–100 bp motifs (≥three repeats) in the nine ciliate macronuclear genomes. The y-axis is log10 transformed.

SSRs are abundant in all macronuclear genomes, accounting for ~7.59% to 11.97% of the whole genome (Table 2; Figure 1). Such abundance is strongly correlated with the genome-wide A/T content (Pearson’s *r* = 0.94, *p* = 0.0002). This confirms that the more polarized the A/T content, the more repetitive the genome. Here, we define a motif as the shortest repeating unit of any given SSR. SSRs with motif sizes 1–10 bp are more abundant than those with longer motifs, especially mononucleotide repeats as homopolymer runs, such as (A)n, (C)n, (G)n, and (T)n (Table 2; Figure 1). In addition to these homopolymer motifs, there are another 166 motifs with sizes of 2–6 bp that are shared in all nine species (Supplementary Table S1). These motifs form similar microsatellite sequences, but their distribution and repeat number do not show specific relevance to each other.

**Figure 2:**
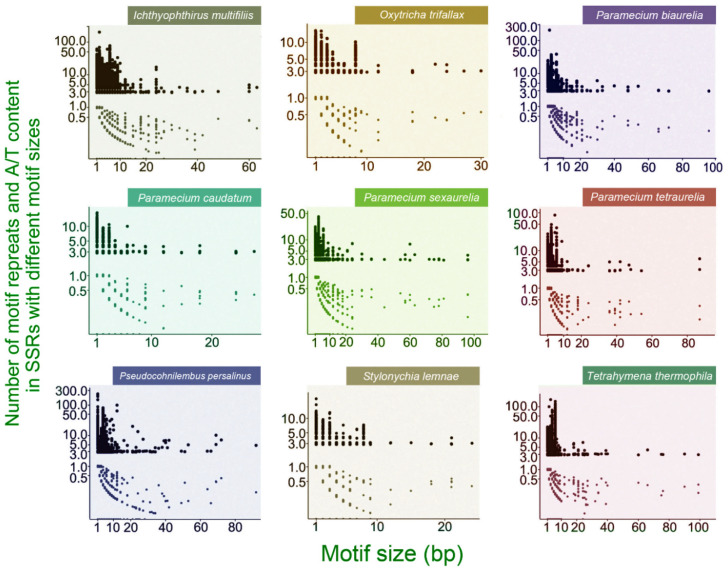
Number of motif repeats, which is represented by y-axis values ≥3, and A/T content in SSRs with different sizes of motifs, represented by y-axis values ≤1. Dots are jittered. Due to the limited jittering-distance, the sizes of dots do not reflect the dominating number of homopolymer SSRs. The y-axis is log10 transformed.

The number of repeats decreases as the motif gets larger (Figure 2). Interestingly, there are peaks at 8-bp motifs in the two spirotricheans, *O. trifallax* and *S. lemnae*, with (G)4(T)4 or (A)4(C)4 at the ends of scaffolds being the majority (50.22% and 70.92%, respectively; Figure 1). These repeat motifs are known telomeric sequences that are added mostly to the ends of the gene-sized chromosomes by telomerases during macronuclear development. However, there are extremely rare internal telomeric repeats, defined as (G)4(T)4 or (A)4(C)4 motifs repeated at least twice in contigs with telomeric repeats at both ends and not located at the first or last 10% of the contigs. In *S. lemnae*, 36 possible internal telomeres are distributed in 36 gene-sized chromosomes; in *O. trifallax*, 39 in 38 chromosomes (Supplementary Table S2). However, the presence of 1000–1500 internal telomeres in the micronuclear polytene chromosomes has been previously reported in *S. lemnae* [40,41]. This indicates that most internal telomeres are eliminated or rearranged during macronuclear development, or unknown internal telomeric sequence difference exists between the macronucleus and micronucleus, as previously reported in *T. thermophila* [42]. In addition, both species have numerous extremely short, gene-sized (i.e., <1 kbp) chromosomes. This is consistent with the assertion that extreme genome fragmentation and amplification increases genome repetivity. By contrast, motifs larger than 10 bp are rare, especially in the two spirotricheans, the assembly scaffolds of which are extremely short (Table 1).

The A/T content of SSRs is significantly higher than that of the corresponding genomes (one-sided paired *t*-test, *t* = -21.563, *df* = 8, *p* = 1.13 × 10^−8^; Table 1 and Table 2) and they are strongly correlated (*r* = 0.90, *p* = 0.0008). The higher A/T content of SSRs is likely due to the dominance of A/T homopolymers in SSRs (Table 2). This domination also elevates the median A/T content of SSRs in all nine species almost to 1.0 (Figure 2). A/T content generally decreases as motif size gets larger (Figure 2; Table 2).

### 3.2. Association between SSRs and Genome Architecture

It is known that repetitive elements contribute to the generation or positional rearrangement of overlapping genes [43,44], for example, in mosquitos the overlapping events are significantly associated with the microsatellite sequences’ amount in the overlapped genes. The microsatellite sequences might have facilitated the crossover events, which lead to positional rearrangement of neighboring genes [44]. Thus, we ask whether ciliate genomes with more SSRs would have more overlapping genes. The proportion of overlapping genes and the proportion of SSRs in the genome are not correlated with each other (Pearson’s *r* = 0.55; *p* = 0.12), giving no significant support to the assertion that SSRs elevate the number of overlapping genes. Nonetheless, the possibility that such lack of correlation is an artifact caused by insufficient annotation quality cannot be excluded. It is noteworthy that there are only three species with overlapping genes and the two with the most overlapping genes, i.e., *Paramecium tetraurelia* and *Tetrahymena thermophila,* have the best-annotated/maintained genomes (Table 1).

**Figure 3:**
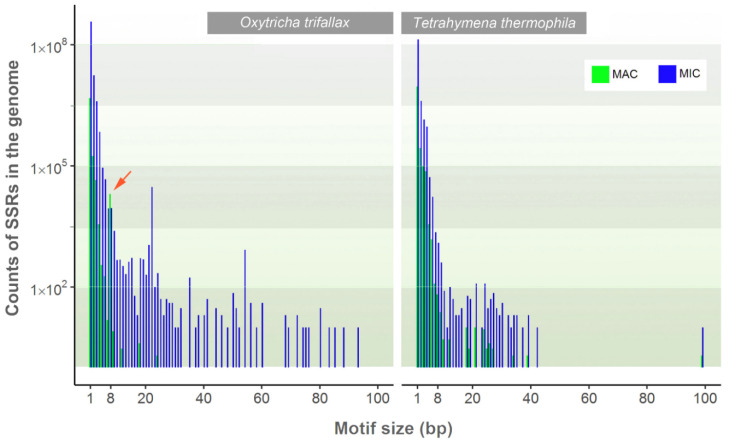
Comparison of SSR counts in the macronucleus and micronucleus of *Oxytricha trifallax* and *Tetrahymena thermophila*. The arrow marks the 8-bp-motif SSRs in the macronuclear genome. The y-axis is log10-transformed.

We also ask the question whether SSRs in the macronuclear and micronuclear genomes follow the same size distributions. Due to the paucity of available micronuclear genomes, only *O. trifallax* and *T. thermophila* are included in this analysis. In *O. trifallax*, for the same motif size, there are more SSRs in the micronuclear genome than in the macronuclear genome, except for those with 8-bp motifs (Figure 3). Of these repeat motifs, 50.22% are in telomeres, probably because the chromosomes are extensively fragmented and amplified during macronuclear development. In *O. trifallax*, 8-bp-motif SSRs account for about 9.46% of all non-homopolymer SSRs in the macronuclear genome, whereas this proportion is only 0.04% in the micronuclear genome. By contrast, in *T. thermophila*, a species with low levels of genome rearrangement, micronuclear SSRs are universally more abundant than the macronuclear SSRs, i.e., there is higher repetivity in the micronuclear than the macronuclear genome (Figure 3).

In order to show more specific SSR patterns, we picked two genes (*MTA6*, *MTB6*; each contains one internally eliminated sequence (IES); NCBI accession numbers: KC405252.1, KC405257.1) in the *T. thermophila* mating type gene family, which are well-studied and have clear gene structural annotations [45]. For each gene, we ran the SSR pipelines and aligned the MDSs (Macronucleus-Destined Sequences) in the micronuclear genome with those in the macronuclear genome (Supplementary Table S3). Consistent with the genome-wide comparison shown in Figure 3, after taking into account all sites of both genes, the macronuclear genes have fewer SSRs than the micronuclear ones. We also parsed out micronuclear intronic SSRs of the two genes and aligned them with those in the macronuclear introns. These conserved SSRs (at least in the two focal genes) do not only include homopolymers such as 5′AAAAAAAA3′, 5′AAAAA3′, but also include microsatellites 5′AATAATAAT3′, 5′ATATAT3′, 5′TATATA3′. The specific functions for these SSRs are unclear, and they could be motifs associated with the rearrangement process. Analyzing SSRs in MDSs shared by both MIC and MAC *MTA6* and *MTB6* genes, we found that ~50% of SSRs have a higher copy number in the macronucleus than in the micronucleus, with the remaining ~50% being equal in the two nuclei. As mentioned above, the total number of SSRs in the two genes (full length) are higher in the micronucleus than in the macronucleus, thus implying that IESs greatly elevate the repetitiveness of the micronuclear genome. This observation from the two genes might be extended to whole-genome-level, although a robust test with fully-annotated macronuclear and micronuclear genomes would be needed. We also found a few SSRs unique to the macronuclear MDSs (i.e., not present in the corresponding MIC genes), for example, 5′CTCCTCCTC3′, 5′CTGCTGCTG3′, 5′GCTGCTGCT3′, 5′TCTCTC3′, 5′TGCTGCTGC3′ in *MTA6*; 5′AACAACAAC3′, 5′AGCAGCAGC3′, 5′AGTAGTAGT3′, 5′CTTCTTCTT3′, 5′GAGAGA3′, 5′TGGTGGTGG3′ in *MTB6* (Supplementary Table S3), suggesting that novel SSRs might be created during the rearrangement process.

Since some tandem repeats with 10–20 bp repeat units are involved in the genome rearrangement [46], we searched SSRs with repeat motifs of 10–20 bases in the micronuclear and macronuclear genomes of both *Tetrahymena thermophila* and *Oxytricha trifallax* (Supplementary Table S4). These SSRs are more abundant in the micronucleus than in the macronucleus (42 in the micronucleus vs. 25 in the macronucleus of *T. thermophila*, and among them 10 are shared with mostly the same sequence and length in both genomes; 368 vs. 8 in *O. trifallax* and 4 are shared; Supplementary Table S4) and are distributed evenly along the scaffolds/chromosomes in both genomes. We also compared these SSRs to those previously published. Interestingly, two identical 19mer SSRs have been detected in two different micronuclear scaffolds (5′ATTATTTCTTTTTACATTT3′; Supplementary Table S4). These are known tandem repeats in Tlr1 [*Tetrahymena* long repeat 1; a member of a gene family with 20-30 DNA elements encoding a polynucleotide transferase; 45], which is involved in genome rearrangement of *T. thermophila* [47] (Supplementary Table S4). This example and the identification of other 10-20bp SSRs confirm the quality of the genomes, the fidelity of the analysis, as well as provide unexplored SSR candidates possibly functioning in the genome arrangement process of both *T. thermophila* and *O. trifallax*.

### 3.3. SSRs in Coding Regions

**Figure 4:**
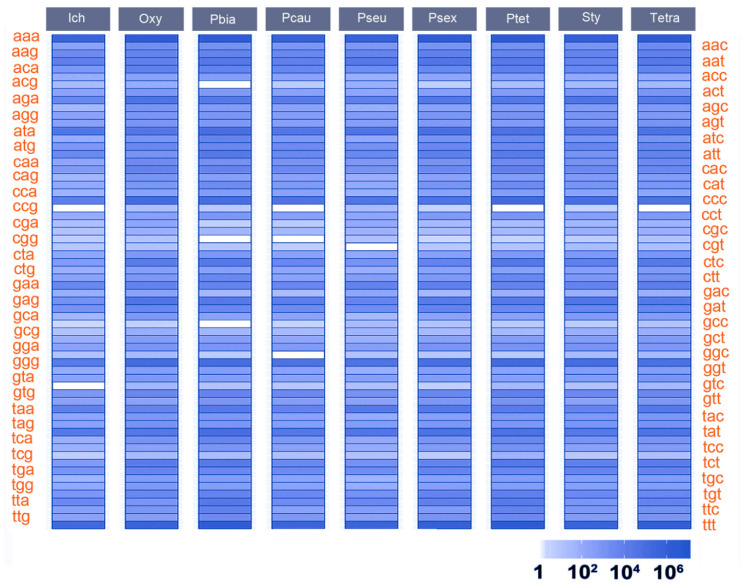
Numbers of codons that are in SSR regions. White boxes represent 0. Ich, *Ichthyophthirius multifiliis*; Oxy, *Oxytricha trifallax*; Pbia, *Paramecium biaurelia*; Pcau, *P. caudatum*; Psex, *P. sexaurelia*; Ptet, *P. tetraurelia*; Pseudo, *Pseudocohnilembus persalinus*; Sty, *Stylonychia lemnae*; Tetra, *Tetrahymena thermophila*.

SSRs are evenly distributed in gene regions, without upstream or downstream biases (Table 2, RPG). As is shown in Figure 4, the top four codons in SSRs of all nine species are AAA (codes for lysine, a charged amino acid), TTT (phenylalanine, a hydrophobic amino acid), GGG (glycine, a hydrophobic amino acid), and CCC (proline, a hydrophobic amino acid). This is consistent with the observation that the vast majority of SSRs are homopolymers.

In order to identify codons that are frequently repeated in coding regions, or possibly most tolerated by the gene, we analyzed codons that are repeated more than 10 times. Isoleucine (hydrophobic), asparagine (hydrophilic), leucine (hydrophobic), tyrosine (hydrophilic), and glutamic acid (charged) codon repetitions are the most abundant in most species. *Ichthyophthirius multifiliis*, *Paramecium biaurelia*, *P. sexaurelia*, and *P. tetraurelia* are the four species with the highest numbers of repeated codons (Table 3). Of the oligohymenophoreans, *P. caudatum* seems to have extremely rare repeated codons. This result suggests that in the four *Paramecium* species included in the present study, the relative abundance of coding SSRs is strongly correlated with genome size (adjusted *R*^2^ = 0.98, *p* = 0.006; Table 1 and Table 2). However, when all nine species were analyzed, the correlation is not significant (adjusted *R*^2^ = 0.13, *p* = 0.19).

## 4. Discussion

In this study, we investigated perfect SSRs in nine ciliate species for which high-quality genomic data are available in order to determine their size distribution, A/T content, repeated codons, and their association with other genomic features. Nevertheless, characterization of SSRs is not the equivalent of a comprehensive investigation of genome repetivity since similar studies have yet to be carried out on large repetitive elements, e.g., transposable elements.

A/T content generally decreases as motif size increases (Figure 2; Table 2), which is consistent with the observation of minisatellites (motif size > 10 bp) being GC-rich in other organisms [48]. In the macronuclear genomes of all the nine ciliates in this study, we also confirm that A/T content of each single motif is also associated with A/T content of the flanking region (the two nucleotides flanking each SSR; Pearson’s *r* ~1, *p* < 2.20 × 10^−16^), which indicates the origin of non-dispersal repeats.

We found that A/T content is strongly associated with SSR abundance. In comparison with other protists, the level of SSR content in ciliates is similar to that of the malaria pathogen *Plasmodium falciparum* (~9% of the genome is SSRs; A/T content 80.67%) [49], while it is much lower than that of *Trypanosoma cruzi* (~30% of the genome is SSRs; A/T content 48.30%) [50], suggesting that the positive correlation between A/T content and SSR abundance is not a general rule in protists, and infers diversifying mechanisms in genome repetitive elements evolution.

Amino acid repeats in proteins are known to play important roles in pathogenesis, cell interaction, motility, cytoskeleton and morphological evolution [13,51,52]. In parasitic ciliates such as *Ichthyophthirius multifiliis* and *Cryptocaryon irritans*, amino acid repeats are important components of the cell surface immobilization antigens (i-ags), which are targets of host antibodies, and codons for amino acids repeats are usually repeated also at the DNA level [53,54,55]. These repeats could cause unequal crossover, creating new alleles and thus increasing antigen diversity. Such recombinogenic expansion of surface antigens might be an adaptive strategy to increase the survival of parasitic ciliates when facing the harsh environment of host secretions. Therefore, the unstable nature of SSRs/tandem repeats could be partially advantageous for ciliate genome evolution, especially for parasitic species.

Across all the ciliate species in this study, the most abundant 3-bp SSRs in coding regions are AAAs, which code for lysines. Lysine-repeats are the most abundant amino-acid repeats in the pellicle alveolins of the alveoli, which are important cellular structures in ciliates for occupying diverse habitats and reflect highly divergent protein evolution [51,56,57,58]. This finding suggests that the SSR motifs are conserved in ciliates with different morphology and life histories. Homopolymers are prone to occur in non-coding regions (Table 2, coding SSR proportion column). It has previously been suggested that homopolymers in non-coding regions can be involved in protein binding, e.g., as upstream promoter elements [59], which implies that the presence of SSRs might be a key factor in driving genome evolution in ciliates. Besides, repeated-codons (>=10 repeats) are rare, potentially as a result of stronger selection against gene mis/dysfunction caused by repetivity in smaller genomes.

In ciliates, the macronucleus is resorbed in each sexual cycle, and its evolution is more driven by epigenetic mechanisms other than classical genetic mechanisms. Relating macronuclear SSRs to the genome evolution of ciliates thus seems to be difficult; however, the macronuclear genome structurally corresponds to the macronucleus-destined sequences in the micronucleus, and the haploid genome sizes of the macronucleus and micronucleus do not usually differ much in most ciliates. In other words, studying macronuclear SSRs’ roles in genome evolution is like an investigation by subsampling the short repetitive elements in the MIC genome (as is shown in Figure 3), with the assumption that short non-IES (internally eliminated sequences) repeats are conserved in both the MAC and MIC, although this might not always be true especially in species with highly fragmented and scrambled genes. Of course, a full picture of SSRs in genome evolution would definitely need the micronuclear genome sequences well annotated in more species.

## 5. Conclusions

This genome-wide and cross-species analysis reveals general features of ciliate SSRs and demonstrates the association between SSRs and the unique genome architectures of ciliates. SSRs might thus be an important driver in genome evolution of this large, charismatic group of microbial eukaryotes.

## Figures and Tables

**Table 1:** Features of macronuclear and micronuclear genomes analyzed in this study.

Species	G (Mbp)	A/T	TNG	n	N50 (kbp)	Platform	Class	Data Source
*Ichthyophthirius multifiliis* (MAC)	48.80	84.09	8096	49	55.11	454, Sanger	Oligohymenophorea	[28]
*Oxytricha trifallax* (MAC)	67.16	68.65	18500	0	3.74	Illumina, 454, Sanger	Spirotrichea	[30]
*Oxytricha trifallax* (MIC)	496.29	71.56	810 ^a^	-	27.81	Illumina, PacBio	Spirotrichea	[35]
*Paramecium biaurelia* (MAC)	79.96	74.23	39242	0	-	Illumina, 454	Oligohymenophorea	[29]
*P. caudatum* (MAC)	30.48	71.80	18509	0	-	Illumina, 454	Oligohymenophorea	[29]
*P. sexaurelia* (MAC)	68.02	75.93	34939	0	-	Illumina, 454	Oligohymenophorea	[29]
*P. tetraurelia* (MAC)	72.09	71.95	39521	144	413	Sanger	Oligohymenophorea	[26]
*Pseudocohnilembus persalinus* (MAC)	55.46	81.19	13186	0	368	Illumina	Oligohymenophorea	[32]
*Stylonychia lemnae* (MAC)	50.16	68.30	20740	0	-	Illumina	Spirotrichea	[25]
*Tetrahymena thermophila* (MAC)	103.01	77.68	24725	60	521	Sanger	Oligohymenophorea	[36]
*Tetrahymena thermophila* (MIC)	157.69	77.92	47 ^b^	-	486.55	Illumina	Oligohymenophorea	[37]

A/T, A/T content of the genome; Class, the taxonomic class in which the species is; G, genome size; MAC, macronucleus; MIC, micronucleus; n, number of overlapping genes; N50, scaffold N50; Platform, genome sequencing platform; TNG, total number of genes in the genome; ^a^, not including internally eliminated sequences (IES)-less genes; ^b^, genes only predicted in non-maintained macronuclear chromosomes, which are lost after macronuclear differentiation.

**Table 2:** Macronuclear simple sequence repeats information.

Species	A/T	SSR/G	H/SSR	A/T-H	*r*1(*P*)	*r*2(*P*)	CSP	RPG(SEM)
*Ichthyophthirius multifiliis*	97.63	11.97	91.04	97.62	−0.72(3.76 × 10^−6^)	−0.55(0.01)	17.08(20.60)	0.50(2.62 × 10^−4^)
*Oxytricha trifallax*	87.74	8.02	95.12	87.76	−0.73(1.27 × 10^−3^)	−0.80(6.08 × 10^−4^)	63.41(70.50)	0.50(1.58 × 10^−4^)
*Paramecium biaurelia*	95.18	8.22	94.52	93.95	−0.19(0.33)	−0.02(0.93)	73.67(72.77)	0.51(1.41 × 10^−4^)
*P. caudatum*	92.17	7.59	95.15	91.86	−0.81(4.51 × 10^−4^)	−0.79(7.10 × 10^−4^)	15.34(86.46)	0.51(2.01 × 10^−4^)
*P. sexaurelia*	95.54	8.68	94.83	95.49	−0.31(0.09)	−0.40(0.05)	69.24(73.43)	0.51(1.97 × 10^−4^)
*P. tetraurelia*	91.97	7.80	94.99	92.07	−0.31(0.15)	−0.08(0.74)	72.24(75.55)	0.50(1.49 × 10^−4^)
*Pseudocohnilembus persalinus*	95.91	11.38	93.75	95.95	−0.48(1.23 × 10^−3^)	−0.56(0.01)	34.59(39.34)	0.50(1.59 × 10^−4^)
*Stylonychia lemnae*	87.35	7.81	94.96	87.39	−0.71(4.76 × 10^−3^)	−0.72(8.67 × 10^−3^)	63.70(71.39)	0.50(1.88 × 10^−4^)
*Tetrahymena thermophila*	96.69	10.09	95.29	96.61	−0.35(0.05)	−0.72(8.67 × 10^−3^)	41.40(49.39)	0.50(1.21 × 10^−4^)

All numbers are percentages, except for those in the r1, r2, and RPG columns. A/T, A/T content of SSRs in the genome; SSR/G, proportion of SSR sequences in the whole genome; H/SSR, proportion of homopolymer runs in SSR sequences; A/T-H, A or T homopolymers out of all homopolymers; *r*1(*P*), Pearson’s correlation coefficient (*P* value) of motif size vs. A/T content at all sites; *r*2(*P*), Pearson’s correlation coefficient (*P* value) of motif size vs. A/T content at coding sites; CSP, coding SSR proportion, proportions of SSRs in coding regions out of all SSRs, proportions of coding sequences out of the whole-genome sequences are in the parentheses; RPG, relative position of homopolymer SSRs in a gene, calculated by (|homopolymer median genomic coordinate-gene start position|+1)/(gene length); SEM, standard error of the mean.

**Table 3:** Total counts of SSRs with codon repeats (>=10) in the nine ciliate genomes.

Codons	Amino Acid	Ich	Oxy	Pbia	Pcau	Psex	Ptet	Pseudo	Sty	Tetra
GCA|GCG|GCC|GCT	Alanine	0	0	0	0	0	0	0	0	0
CGA|CGG|CGC|CGT|AGA|AGG	Arginine	8	0	0	0	5	0	1	0	1
AAC|AAT	Asparagine	65	0	70	0	111	38	8	0	12
GAC|GAT	Aspartic acid	13	0	0	0	1	3	2	0	1
TGC|TGT	Cysteine	1	0	1	0	0	1	0	0	0
GGA|GGG|GGC|GGT	Glycine	1	1	1	1	2	1	1	1	1
GAA|GAG	Glutamic acid	16	0	1	1	6	5	7	0	3
CAA|CAG	Glutamine	0	0	1	0	1	3	1	0	0
CAC|CAT	Histidine	4	0	0	0	0	0	0	0	0
ATA|ATC|ATT	Isoleucine	80	1	70	1	113	20	6	1	6
CTA|CTG|GTC|CTT|TTA|TTG	Leucine	13	0	98	0	0	48	1	0	2
AAA|AAG	Lysine	15	0	5	0	10	1	10	0	5
ATG	Methionine	2	0	0	0	1	0	3	0	1
TTC|TTT	Phenylalanine	2	0	5	0	0	0	2	0	0
CCA|CCG|CCC|CCT	Proline	1	0	1	0	0	5	1	0	0
TCA|TCT|TCC|TCT|AGC|AGT	Serine	4	0	2	0	0	0	0	0	0
ACA|ACG|ACC|ACT	Threonine	10	0	1	0	4	3	2	0	1
TGG	Tryptophan	2	0	0	0	0	0	0	0	1
TAC|TAT	Tyrosine	17	0	60	0	0	30	0	0	0
GTA|GTG|GTC|GTT	Valine	3	0	0	0	1	0	3	0	1

Ich, *Ichthyophthirius multifiliis*; Oxy, *Oxytricha trifallax*; Pbia, *Paramecium biaurelia*; Pcau, *P. caudatum*; Psex, *P. sexaurelia*; Ptet, *P. tetraurelia*; Pseudo, *Pseudocohnilembus persalinus*; Sty, *Stylonychia lemnae*; Tetra, *Tetrahymena thermophila*.

## Supplementary Materials

The following are available online at https://www.mdpi.com/2076-2607/8/5/662/s1, Table S1. The SSR motifs that are shared in all nine ciliate species in this study; Table S2. Details of internal telomeric repeats in *Stylonychia lemnae* and *Oxytricha trifallax*. Table S3. The SSRs from the *MTA6* and *MTB6* genes in the macronucleus and micronucleus of *Tetrahymena thermophila*. Table S4. Details of the macronuclear and micronuclear SSRs with motif size 10–20 bp in *Tetrahymena thermophila* and *Oxytricha trifallax*.
